# Developmental regulation of long-range neuroblast migration by Eph/ephrin signaling

**DOI:** 10.3389/fnins.2025.1670635

**Published:** 2025-10-08

**Authors:** Daria Yeroshenko, Chamalka de Silva, Anika Burli, Sarah Bellizzi, Vijender Singh, Joanne Conover

**Affiliations:** ^1^Conover Laboratory, Department of Physiology and Neurobiology, University of Connecticut, Storrs, CT, United States; ^2^Computational Biology Core, University of Connecticut, Storrs, CT, United States

**Keywords:** Eph receptor tyrosine kinases, rostral migratory stream (RMS), postnatal neurodevelopment, single-cell RNA analysis, neural stem/progenitor cells, postnatal migration

## Abstract

In the developing mouse anterior forebrain, the rostral migratory stream (RMS) supports continued proliferation and efficient transportation of large quantities of neuroblasts from the ventricular-subventricular (V-SVZ) stem cell niche to the olfactory bulb (OB). Astrocytes aid this migration by providing a glial network through which chains of fasciculated neuroblasts move. The largest receptor tyrosine kinase family, Eph receptors, and their ephrin ligands have been implicated in controlling neuroblast migration and astrocyte organization within this pathway. However, a clear understanding of the regulatory mechanisms underlying Eph/ephrin signaling remains elusive due, in part, to the complexity of heterogeneous expression patterns in both neuroblasts and astrocytes, as well as the cytoarchitectural changes that occur during postnatal development. To address this gap, we analyzed RMS cytoarchitecture together with transcriptomic and proteomic profiles at postnatal days P6, P12, and P60, and mapped Eph-ephrin interactions using predictive interaction models. Our data revealed temporally regulated, cell type-specific, receptor-ligand interactions, highlighting the prevalence and dynamic shifts of neuroblast-neuroblast, neuroblast-astrocyte, astrocyte-astrocyte interactions. Together, these findings established a framework that deconvoluted and characterized Eph and ephrin signaling as the RMS changed from a diffuse stream of migratory neuroblasts to a highly constricted pathway of neuroblast chains within astrocytic networks.

## 1 Introduction

Cell migration is a highly controlled, essential process in the development and maintenance of a healthy brain. Mammals, from humans to mice, retain a postnatal neuronal migratory pathway, the rostral migratory stream (RMS), originating in the ventricular-subventricular zone (V-SVZ) along the lateral ventricles and ending in the olfactory bulb. An additional pathway, the medial migratory stream (MMS), found in humans, extends from the V-SVZ to the ventromedial prefrontal cortex (Paredes et al., 2016; Sanai et al., 2011). In mice, the RMS persists into adulthood and old age (Mobley et al., 2013) based on continual V-SVZ neurogenesis supplying new neuroblasts for migration (Apostolopoulou et al., 2017; Obernier and Alvarez-Buylla, 2019; Shook et al., 2012; Sun et al., 2010). While in humans, V-SVZ neurogenesis is greatly reduced by 2 years of age along with accompanying depletion of migratory neuroblasts in both the RMS and MMS (Sanai et al., 2011; Sorrells, 2024). The well-conserved cellular architecture of the RMS in mouse and human (Sanai et al., 2011) provides the impetus for understanding RMS regulatory control.

Developmental assessment reveals that prior to birth (E16–E18) the RMS emerges as a rostral extension of the lateral ventricle, forming a continuous path toward the olfactory bulb (Pencea and Luskin, 2003; Peretto et al., 2005). During embryonic and neonatal development, loosely aggregated neuroblasts migrate collectively along this extension (Nie et al., 2010; Pencea and Luskin, 2003). Observations at P6 indicate that astrocytes are found mainly along the periphery of the RMS with only a few integrating within the pathway. By P12, astrocytes are integrated with the migratory neuroblasts within the RMS core (Peretto et al., 2005; Todd et al., 2017). Thereafter, the RMS consists of tightly fasciculated chains of neuroblasts that migrate through a dense network of astrocytes and a sparse, parallel array of blood vessels (Khodosevich et al., 2013; Meller et al., 2023; Sun et al., 2010).

Known regulators of RMS organization and neuroblast migration include PSA-NCAM, which helps mediate neuroblast-neuroblast contact, facilitating chain migration (Battista and Rutishauser, 2010; Sun et al., 2010), and neurotransmitters such as GABA which modulate migratory speed of neuroblasts through the GAT4 transporter on astrocytes (Sun et al., 2010). While growth factors such as VEGF promote neuronal migration through VEGF receptor 1 on astrocytes (Wittko et al., 2009) and BDNF, secreted by endothelial cells, supports entry into migratory phases for neuroblasts (Bressan and Saghatelyan, 2021; Khodosevich et al., 2013). Additionally, calcium-binding protein SCGN facilitates neuroblast migration by triggering calcium-dependent MMP-2 release leading to extracellular matrix remodeling (Hanics et al., 2017) and repulsive ligand-receptor pairs, such as Slit-Robo, help guide neuroblast migration within the astrocytic meshwork, with SLIT1 secreted by neuroblasts binding ROBO2 receptors on astrocytes, repelling astrocytic processes (Bressan and Saghatelyan, 2021; Kaneko et al., 2010). Other known regulators include uni-directional and bi-directional signaling through the Eph receptor tyrosine kinases and their ligands, the ephrins. Eph-ephrin signaling functions in cell proliferation and migration (Conover et al., 2000; Dixon et al., 2016; Holmberg et al., 2005; Khodosevich et al., 2011), with EphA4 signaling required for compact and directional organization of neuroblasts and astrocytes within the mature RMS (Todd et al., 2017).

Eph-ephrin signaling has known roles in directional cell migration and patterning of the brain and other organ systems (Coulthard et al., 2012; Klein, 2012; Taylor et al., 2017) and, like these systems, regulatory control within the RMS presents a particularly complex challenge since multiple Ephs and ephrins are expressed on both neuroblasts and astrocytes within the RMS (Todd et al., 2017). Eph receptors and ephrin ligands make up the largest family of receptor tyrosine kinases. There are nine EphA (1–8, 10) and five EphB (1–4, 6) receptors each capable of binding to complementary ephrin ligands within their subclass to initiate signaling. Although binding typically occurs within the specific A or B subclasses, crosstalk and redundancy can occur within the classes, adding to the complexity of Eph/ephrin signaling and making knockout/knockdown studies difficult to design and interpret (Rasool and Jahani-Asl, 2024; Taylor et al., 2017). Since Ephs and ephrins are cell surface receptors and ligands, signaling is typically dependent on cell–cell contact, and as a unique feature, both ligands and receptors can signal upon activation through binding. Bi-directional signaling, where signaling through the Eph receptor is “forward” signaling, while signaling through the ephrin ligand is “reverse” signaling (Taylor et al., 2017), allows response by both contacting cells. In addition to cell–cell contact, Eph/ephrin signaling can occur through exosomes (Gong et al., 2016), proteolytic cleavage (Atapattu et al., 2014), ECM tethering (Jülich et al., 2009), cis-inhibition (Cecchini and Cornelison, 2022), and sustained signaling via endocytosis (Valenzuela and Perez, 2020). We, previously, found that EphA4 is required for RMS organization (Todd et al., 2017). By P12, EphA4^−/−^ mice showed disorganization of the RMS astrocytic meshwork, loss of neuroblast fasciculation, and aberrant neuroblast migration outside of the normal RMS boundaries. Additionally, RNA analysis of RMS cells revealed that *EphA4* receptors and *Efn* (ephrin) ligands exhibit heterogeneous expression profiles in both astrocyte and neuroblast populations, with *EphA4* expressed in 40% of both cell types. However, to date, no comprehensive developmental framework has been established detailing the range of Eph/ephrin permutations or possible receptor-ligand interactions within the RMS.

Here, we address these gaps in our understanding of potential Eph-ephrin interactions associated with long-range neuroblast migration and the role they may play during developmental changes within the RMS. Characterization of cytoarchitectural organizational changes to the RMS at P6, P12, and P60 together with single-cell RNA sequencing (scRNA-seq) of the micro-dissected RMS allowed us to resolve temporally regulated expression patterns of Ephs and ephrins across neuroblast subpopulations. Validation of activated (phosphorylated) Ephs and ephrins in tissue slices together with predictive interactional modeling (CellChat) further allowed us to infer specific signaling pairs, their directionality, and temporal restrictions. Together, we integrated single-cell transcriptomics, signaling inference tools, with phosphorylation status to define Eph/ephrin signaling modes within the RMS. Our studies illustrate how these computational approaches can help to map a complex, dynamic molecular landscape regulating RMS organization and maturation.

## 2 Materials and methods

### 2.1 Mice

Male and female CD-1 mice were housed on a 12-h day-night cycle with free access to water and food. Mice were treated according to the guidelines from the University of Connecticut, Institutional Animal Care and Use Committee (IACUC) and the NIH. Sample replications for each experiment are described in the relevant method subsections listed below. Equal numbers of male and female mice were used for each experiment and any sex differences are reported in the Results section when present.

### 2.2 Immunofluorescence staining

Both male and female mice were used for immunostaining experiments for the ages listed and then combined when no significant difference was observed between sexes. Mice were anesthetized with isoflurane and then perfused intracardially with 0.9% saline. After an overnight post-fixation in 4% paraformaldehyde (PFA) at 4 °C, brain tissues were cut into 50 μm coronal or sagittal sections with a vibrating microtome. Brain sections were blocked with 0.1% Triton X-100 and 10% donkey serum (NDS, Jackson ImmunoResearch, AB_2337258) in PBS (1X) for 1 h at room temperature, followed by overnight incubation at 4 °C with the primary antibodies (Supplementary Table 1). The next day, sections were rinsed and incubated with Alexa Fluor secondary antibodies (Supplementary Table 2). The antibodies used for immunostaining are described in the Supplementary material. Following primary and secondary antibody staining, tissue samples were washed for 10 min in PBS (1X) and then incubated with DAPI (10 mg/mL, Thermo Fisher Scientific, RRID:AB_2629482) for 10 min. DAPI solution was removed followed by a final three rinses with PBS (1X) before the tissue was placed onto slides and cover-slipped using Aqua-Poly/Mount (Polysciences Inc., 18606-20).

### 2.3 5-Ethynyl-2-deoxyuridine

To label newly divided migratory neuroblasts, 5-Ethynyl-2-deoxyuridine (EdU) (100 mg/kg, from a 10 mg/mL stock solution) was injected intraperitoneally 3 days before the mouse brain collection dates (i.e., P3 for P6, P9 for P12, and P57 for P60 collection timepoints). Mice were perfused with 0.9% saline. Brains were removed and postfixed in 4% PFA overnight followed by three 5-min washes in PBS (1X). Brains were sectioned coronally (50 μm) and after primary and secondary antibody staining, EdU was visualized using the Click-It EdU Alexa Fluor-488 Imaging Kit (Thermo Fisher Scientific, C10337) according to the manufacturer’s instructions.

### 2.4 Image acquisition and analysis

Confocal images of sequential brain sections were captured at 40X on a Leica SP8 confocal microscope with Leica Application Suite X (LAS X) software as either z-stacks or single plane images. Images were processed using ImageJ (National Institutes of Health). Coronal sections of the horizontal arm of RMS beginning at 2.3 mm and ending at 2 mm relative to bregma were analyzed using ImageJ Cell Counter to obtain the number of EdU^+^, DCX^+^ and either phospho-EphA3/4/5, phospho-EphA7, phospho-EphB1/2, or phospho-ephrinB1/2/3 positive cells (*n* = 3 images/RMS/hemisphere/animal; *n* = 6 animals/age group). Same coronal sections were assessed for GFAP^+^ co-expression with phosphorylated antibodies. To quantify RMS astrocyte coverage and marker co-expression, the RMS area was isolated and GFAP channel and Eph or ephrin channels were binarized in ImageJ using Threshold function. Co-expression was visualized using ImageJ’s Image Calculator and area of astrocytes and astrocyte co-expression were recorded using Measure function (*n* = 3 images/RMS/hemisphere/animal; *n* = 6 animals/age group). Percentages of co-expressing cells were determined and data were visualized using boxplots and statistical comparisons between ages were performed using pairwise Wilcoxon rank-sum tests.

RMS area (μm^2^) was measured on coronal sections of the RMS beginning at 2.3 mm and ending at 2 mm relative to bregma, by outlining the RMS visualized by GFAP and DCX staining, using the ImageJ Measure tool; total EdU^+^, DCX^+^ cell counts per 250 μm were also counted using ImageJ Cell Counter (*n* = 5 images/RMS/hemisphere/animal; *n* = 6 animals/age group). Area measurements were averaged for each mouse and the mean RMS area per mouse and total EdU^+^, DCX^+^ cell counts per 250 μm were compared across ages (P6, P12, P60) using boxplots and unpaired two-tailed *t*-tests. A correlation between area and cell counts was performed by calculating mean RMS area and mean EdU^+^, DCX^+^ cell counts per slice for each mouse. Average values were plotted with each point representing a single mouse. Density was calculated using the total EdU^+^, DCX^+^ cell counts and the summed RMS area measurements. RMS volume was estimated as area multiplied by slice thickness (50 μm) and normalized per 10^6^ μm^3^. Volume-normalized densities were visualized and compared using boxplots and *t*-tests. Sex-based differences were assessed using boxplots and unpaired two-tailed *t*-tests. All visualizations and statistics were performed in RStudio.

### 2.5 Single-cell RNA-seq sample preparation and sequencing

For the P6 and P12 ages, mice (two males and two females for each timepoint) were anesthetized with isoflurane and then perfused intracardially with 0.9% saline. Brains were removed and the RMS was carefully micro-dissected from the rest of the brain. The RMS is a relatively narrow pathway; therefore, our data likely includes some cell types from regions immediately adjacent to the RMS. RMS tissue was dissociated according to the 10X Genomics Chromium sample preparation protocol. Tissue was cut into smaller pieces and placed into 2 mL papain solution (BrainBits PAPHE, NC0435282). Tissue was incubated at 37 °C for 20 min, the papain solution was removed and 2 mL of Hibernate E medium (BrainBits HE500, NC9063748) was added to the samples and the sample was triturated with a fire-polished 9-inch Pasteur pipette. The sample was then set aside for 1 min to allow for the tissue debris to settle. Cells in the supernatant were removed and passed through a 70 μm mesh filter (Electron Microscopy Sciences, 6475200) two times. Cells were centrifuged for 2 min at 200rcf., the supernatant discarded, the cells resuspended in 1 mL of NbActiv1 medium (BrainBits NB1100, NC1482275) and passed through a 70 μm mesh filter one final time before being placed on ice. Cells were stained with DAPI (1 μg/mL) for 5 min and live (DAPI-negative) singlets were sorted using Aurora Cell Sorter (Cytek) with a 100 μm nozzle at low pressure. Sorted live cells were collected into NbActiv1 medium (BrainBits NB1100, NC1482275). For our P60 sample, the same harvesting and microdissection procedures were followed. However, tissue dissociation was performed using the Singulator 200 (S2 Genomics) following the manufacturer’s protocol (Cell Isolation from Fresh Tissue for Single Cell Sequencing Applications, S2 Genomics). Cell viability and concentration were assessed using a Bio-Rad TC20 Automated Cell Counter and loaded onto a 10X Genomics Chromium System. Libraries were generated with the 10X Chromium Single Cell 3′ v2 reagent kit according to the manufacturer’s instructions and sequenced on an Illumina NovaSeq 6000.

### 2.6 Single-cell RNA-seq data analysis

Sequencing data was processed using Cell Ranger (v9.0.1). FASTQ files were generated with the cellranger mkfastq command and aligned to the mouse reference genome (mm10) using cellranger count. The resulting gene-barcode matrices were used for downstream quality control and analysis. Data normalization, analysis, and visualization were performed using Seurat software (v5.1.0). Low quality cells were filtered by excluding those based on <500 or >6,000 detected genes and >10% mitochondrial content (Luecken and Theis, 2019). Data was log normalized, merged, and batch correction and integration were performed using Seurat’s anchor-based canonical correlation analysis method. Principal component analysis was performed on variable features, then clustered to generate UMAP graphs. Cell identities were determined by finding cluster-enriched genes and comparing them to established cell-type specific expression profiles. To infer sex of individual cells, a module score was calculated using expression of Y chromosome-linked genes (*Uty*, *Kdm5d*, *Ddx3y*) with AddModuleScore function. Cells with a positive score were classified as male, and the remaining cells were classified as female. DotPlot function was used for visualization of selected genes and split dotplots were generated using the split.by parameter to compare the gene expressions across different conditions (age and sex). To create an annotated DotPlot, average expression and percentage of cells expressing Ephs and ephrins were extracted from a previously created DotPlot. Then this data was visualized as a scatterplot using ggplot and the threshold of genes with an average expression greater than 0.5 and detected in more than 25% of cells was annotated using geom_text.

Differential gene expression analysis between clusters was performed using FindMarkers function. Genes were categorized as upregulated or downregulated based on the adjusted *p*-value threshold of 0.05 and an absolute log_2_ fold change >0.6. A volcano plot was generated in ggplot2 with the top 10 most upregulated and downregulated genes. Cluster proportions and specific cluster counts were extracted from the Seurat object metadata and visualized using ggplot. Cell–cell communication was characterized using the CellChat (v2.1.2) R package. Interactions involving EphA6 were not present in CellChatDB.mouse but because we observed EphA6 RNA and protein expression in neuroblasts, we manually added EphA6-ephrinA5 interaction based on structural homology with other EphA receptors and previously reported overlapping spatial expression (Deschamps et al., 2010). To examine interactions among astrocyte and neuroblast clusters, circle plots and bubble plots were generated using netVisual_circle and netVisual_bubble. All predicted interactions were visualized for EPHA and EPHB pathways. For the annotated bubble plot, geom_text function was used with a threshold of <0.01. Radar Plots were created using the fmsb software package, where the RNA gene expression percentages were extracted for merged neuroblast and merged astrocyte clusters. Phosphorylation percentages were taken from the EdU coexpression analysis described above. The extracted values were combined into radar plots for each age (P6, P12, and P60) and cell type (neuroblasts, astrocytes).

## 3 Results

### 3.1 RMS cell composition and organizational changes during development

**Figure 1:**
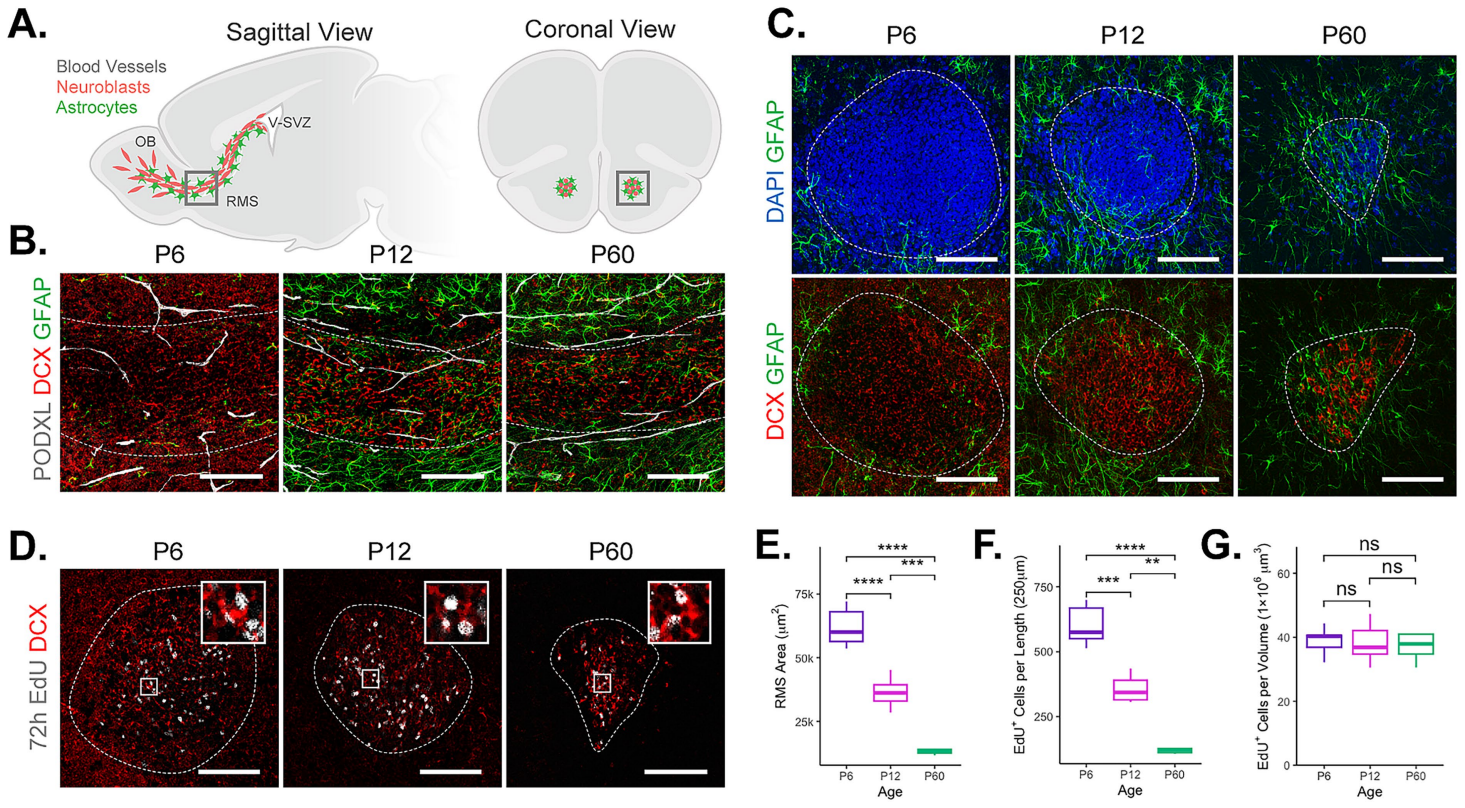
RMS cellular architecture characterized at different developmental stages. (**A**, left) Sagittal view of P60 mouse brain shows neuroblasts (red) born in the V-SVZ of the lateral ventricle migrate through the RMS supported by a meshwork of astrocytes (green) and blood vessels (white). Upon entering the OB, neuroblasts switch their migration from tangential to radial. (**A**, right) Schematic of a coronal mouse brain section with neuroblasts (red) and astrocytes (green). **(B)** Representative confocal images of sagittal sections of the RMS labeled with DCX (red), GFAP (green), PODXL (white) for the indicated ages. Dotted, white line outlines the RMS. **(C)** Representative confocal images of coronal sections through the horizontal arm of the RMS showing DCX^+^ neuroblasts (red), GFAP^+^ astrocytes (green), and DAPI^+^ nuclei (blue) for the indicated ages. White dotted line outlines the RMS. **(D)** Representative confocal images of the RMS (coronal sections) showing proliferative co-labeled EdU^+^ DCX^+^ neuroblasts (red, white) for the indicated ages. Magnified square demonstrates co-labeling of EdU^+^ DCX^+^ neuroblasts (size: 25 × 25 μm). **(E)** Quantification of RMS cross-sectional area across ages. **(F)** Quantification of total EdU^+^ cells per 250 μm length of the RMS across ages. **(G)** Quantification of EdU^+^ cell density per 1 × 10^6^ μm^3^ RMS volume across ages. Scale bars = 100 μm. Pairwise comparisons were performed using unpaired, two-sided Student’s *t*-tests. Significance is indicated as follows: *p* ≤ 0.01 (**), *p* ≤ 0.001 (***), *p* ≤ 0.0001 (****), ns (not significant).

Large quantities of newly generated neuroblasts migrate approximately 5 mm through the adult RMS (Figure 1A) (Mobley et al., 2013; Poon and Goldowitz, 2014). This pathway is efficient and organized, but the organization changes over the course of development (Meller et al., 2023; Peretto et al., 2005; Poon and Goldowitz, 2014; Todd et al., 2017). To build upon previous findings and establish cell compositional and spatial organizational changes within the developing RMS, we first examined the distribution of neuroblasts, astrocytes, and endothelial cells in coronal and sagittal forebrain sections at three developmental timepoints P6, P12, and P60. At P6, DCX^+^ neuroblasts form a broad stream with a large cross-sectional area (Figures 1B,C: sagittal and coronal view, respectively) with GFAP^+^ astrocytes mainly concentrated around the RMS periphery, only a few infiltrated within the RMS core. Endothelial cells (PODXL^+^) lining blood vessels were organized in parallel with migratory neuroblasts; this organization, while sparse, remained relatively consistent across all ages examined (Figure 1B). By P12, the RMS was more compact (Figures 1B,C). DCX^+^ neuroblasts occupied a narrower cross-sectional area, and astrocytes at this stage were found within the RMS core, creating an early formation of the glial network that aids neuroblast migration (Lois et al., 1996; Meller et al., 2023; Todd et al., 2017). By P60 (young adult), the RMS was condensed, with fasciculated bundles of DCX^+^ neuroblasts migrating through a tightly organized, intricate meshwork of astrocytes (Figures 1B,C; Meller et al., 2023; Sun et al., 2010). As mentioned, the endothelial vasculature remained stable, paralleling neuroblast migration within the RMS. Quantification of the RMS cross-sectional area (Figure 1E) confirmed a progressive reduction from P6 to P60, consistent with RMS anatomical development.

EdU labeling (72 h) was used to assess changes in the number of newly generated, migratory neuroblasts at each timepoint within a defined region of the RMS (Khodosevich et al., 2013; Figures 1D,F,G). The total number of EdU^+^ neuroblasts within a defined 250 μm length of RMS (horizontal arm, see schematic Figure 1A) decreased significantly with age (Figure 1F). However, cell density of EdU^+^ neuroblasts per 1 × 10^6^ μm^3^ volume of RMS remained constant across the ages (Figure 1G), confirming the direct relationship between reduced neuroblast number and contraction of RMS (Figure 1E), as expected. Together, our data indicate that as the mouse ages, the RMS undergoes structural refinement with the addition of a dense astrocytic network, promoting a more streamlined and spatially restricted pathway that accompanies scaled down neurogenesis (Mobley et al., 2013). Quantitative analyses, including structural changes, revealed no differences between the sexes in RMS area, neuroblast counts, or neuroblast density (Supplementary Figures 1A–C).

### 3.2 scRNA-seq reveals distinct neuroblast and astrocyte subpopulations in the developing RMS

**Figure 2:**
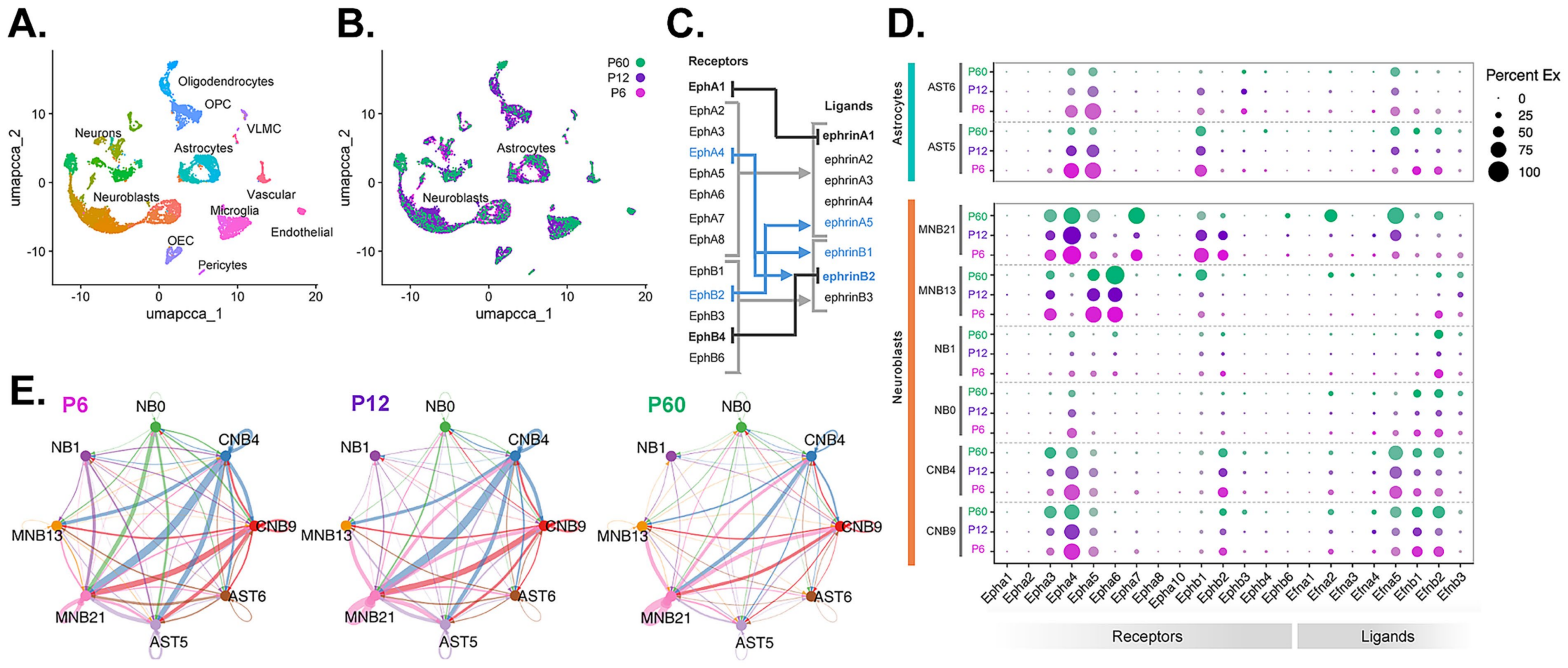
Neuroblasts and astrocytes exhibit distinct Eph/ephrin profiles and cell–cell communication profiles. Single-cell RNA-seq analysis of the micro-dissected RMS from P6, P12, and P60 mouse brains. **(A)** Uniform Manifold Approximation and Projection (UMAP) plot of scRNA-seq cell types captured after demultiplexing, doublet removal, and clustering. Abbreviations: oligodendrocyte progenitor cells (OPC), vascular leptomeningeal cells (VLMC), olfactory ensheathing cells (OEC). **(B)** UMAP plot of cell clusters grouped by age: P6 (pink), P12 (purple), and P60 (green). **(C)** Schematic representation of binding affinities of Eph receptors and ephrin ligands. Arrows and brackets represent binding within the same class (light gray), cross-class binding (blue), exclusive binding (black) modified from Gerstmann and Zimmer (2018). **(D)** Age split DotPlot of Eph/ephrin expression in RMS neuroblast [cycling neuroblasts CNB9, 4; neuroblasts (NB0, 1), maturing/mature neuroblasts (MNB13, 21)] and astrocyte (AST5, 6) clusters. **(E)** Circle plots showing inferred aggregate Eph and ephrin signaling networks between neuroblast and astrocyte clusters. Arrow thickness represents communication strength, colors indicate sending cell types.

Heterogeneous neural stem cell (NSC) populations from spatially distinct regions along the V-SVZ produce new neurons, neuroblasts, that organize into chains (Fiorelli et al., 2015; Obernier and Alvarez-Buylla, 2019) for continued, long-range migration through the RMS to their final site of differentiation within the OB. Once in the OB, neuroblasts disperse radially and are thought to establish within specific locations based on their predetermined neuronal subtype established in the V-SVZ (Batista-Brito et al., 2008). We used single-cell RNA transcriptomics to investigate the cellular heterogeneity of both RMS neuroblasts and astrocytes over developmental time (P6, P12, and P60). We profiled single cells from the micro-dissected RMS of CD-1 mice at P6, P12, and P60. Cells from each age were merged using canonical correlation analysis to account for batch effects. Unsupervised clustering based on principal component analysis of the transcriptional profiles was performed and visualized using UMAP (Figure 2A). Cell cluster identities were annotated based on the detection of known cell type markers (Supplementary Figure 2A). A joint UMAP of all cells depicted based on sample age showed cell distribution across clusters, indicating successful integration and minimized age-based batch effects (Figure 2B).

We identified a total of 27 cell clusters, with six clusters corresponding to neuroblasts and three to astrocytes. Differential analysis of the three astrocyte clusters (AST5, 6, 11) revealed specific molecular identities (Supplementary Figures 3A–C). AST5 exhibited upregulated expression of *Thbs4* (Supplementary Figure 3D), which is a gene associated with extracellular matrix remodeling and is known to be expressed in RMS astrocytes (Girard et al., 2014). AST6 exhibited upregulated *Agt*, which suggests potential vascular functions, and *Rarres1*, which is involved in differentiation and cell adhesion. AST11 displayed upregulated *Gabbr2* implicating GABAergic signaling and ion homeostasis and downregulated *Ezr*, *Vim*, *Hes5*, *Mfge8*, which were all present in AST5 and AST6. Together, these data suggested that AST5 and AST6 are likely RMS astrocytes, while AST11 likely identifies homeostatic astrocytes in RMS-adjacent regions (Cebrian-Silla et al., 2025; Endo et al., 2022; Zeisel et al., 2018). Additionally, proportionally AST11 made up the smallest percentage of astrocytes across ages (Supplementary Figures 3E,F) and displayed minor signaling contribution with neuroblasts (see Supplementary Figure 5). Based on this, AST5 and AST6 were used for further analyses of RMS interactions.

Expression profiling of selected genes in the six neuroblast clusters (CNB9, 4; NB0, 1; MNB13, 21) (Supplementary Figure 3G) identified proliferative neuroblast clusters (CNB9, 4) based on upregulated cell cycle genes (e.g., *Mki67*, *Top2a*); immature neuroblast clusters (NB0, 1) based on developmental and migratory genes (e.g., *Dcx*, *Tubb3*); intermediate maturing neuroblasts (MNB13) based on neurodevelopmental genes (e.g., *Grm8*, *Camk4*); and later stage neuroblasts (MNB21) based on genes indicating neuronal maturation (e.g., *Neurod1*, *Rbfox3*). Maturing and mature late-stage neuroblast populations MNB13 and MNB21, respectively, exhibited different markers linked to V-SVZ spatial origin (Cebrian-Silla et al., 2021; Fiorelli et al., 2015). Specifically, MNB13 neuroblasts have a higher expression of *Dlx1*/*2* and *Sp8* which are known to be linked to the lateral V-SVZ, suggesting that MNB13 neuroblasts are likely to become GABAergic interneurons (Calbindin^+^ and TH^+^). MNB21 neuroblasts have a higher expression of *Emx1*, *Nfix*, *Eomes* which is linked to the dorsal SVZ (Fiorelli et al., 2015; Kohwi et al., 2007; Obernier and Alvarez-Buylla, 2019), suggesting that MNB21 neuroblasts are more likely to become glutamatergic interneurons (Calretinin^+^). Other neuroblast clusters (CNB9, 4: NB0, 1) contain cells that express either dorsal, lateral, or a mix of both sets of markers, suggesting possible lineage progression from neural stem cells to mature neuroblasts. Although proportions of immature and proliferative neuroblast clusters remain relatively consistent between the three ages, intermediate (MNB13) and later stage neuroblast (MNB21) percentages decreased with age (Supplementary Figure 3H), suggesting delayed differentiation onset with neuroblasts remaining immature longer. These changes are within the context of an overall decrease of neuroblast percentage from P6 to P60 (≈53%) (Supplementary Figure 3I).

### 3.3 Neuroblast subclusters exhibit distinct Eph/ephrin profiles, while RMS astrocyte subclusters share uniform Eph/ephrin profiles

To investigate cell-specific expression patterns and to define putative cell–cell interactions based on Eph and ephrin signaling within the RMS, we first generated a DotPlot of all Eph receptors and ephrin ligands arranged by age. Eph receptors and ephrin ligands bind preferentially within groups (A or B) but there are some exceptions (EphA4, EphB2) that are capable of binding across classes (Figure 2C, adopted from Gerstmann and Zimmer, 2018; Charmsaz et al., 2017; Coulthard et al., 2012; Gerstmann and Zimmer, 2018; Pasquale, 2004). To identify robust and biologically significant gene markers, we generated a scatterplot of the average expression level versus the percentage of cells expressing each gene across all clusters (Supplementary Figure 4A). This plot allowed us to look at the overall distribution of gene expression and determine appropriate thresholds to minimize the inclusion of low-confidence or background signals (Stuart et al., 2019). We defined thresholds based on the distribution, selecting genes with an average expression greater than 0.5 and detected in more than 25% of cells in at least one cluster. These thresholds highlight Eph and ephrin genes that are both sufficiently expressed and broadly present (Supplementary Figure 4B). Proliferative neuroblast clusters (CNB9, CNB4) had receptors *EphA3*, *EphA4*, *EphA5*, *EphB2* and ligands *Efna5*, *Efnb1*, *Efnb2* meeting the thresholds, while immature neuroblast clusters (NB0, NB1) mainly had *EphA4*, *Efnb1*, and *Efnb2*. Intermediate maturing neuroblasts (MNB13) had statistically meaningful expression of *EphA3*, *EphA5*, *EphA6* and *EphB1*, while later stage neuroblast cluster (MNB21) had *EphA3*, *EphA4*, *EphA5*, *EphA7*, *EphB1*, *EphB2* and *Efna5* (Figure 2D). These data suggest that neuroblast subclusters have specific Eph and ephrin expression signatures that change based on the neuroblasts’ developmental stage. In contrast, when we analyzed astrocyte subclusters, we found that RMS astrocytes (AST5 and AST6) expressed receptors *EphA4*, *EphA5*, *EphB1*and ligands *Efna5*, *Efnb1*, and *Efnb2* (Figure 2D) suggesting that RMS astrocytes might be involved not just in EphA4 signaling but also interactions involving EphA5, EphB1, ephrinA5, ephrinB1, and ephrinB2. However, the overall complexity of *Eph*/*Efn* expression and the potential for binding promiscuity require more in-depth analysis of cell–cell interactions.

To evaluate whether Eph/ephrin expression differed by sex, cells were classified as male or female based on the expression of the Y-chromosome linked genes (i.e., *Uty*, *Kdm5d*, *Ddx3y*). UMAP visualization showed even distribution of male and female cells across all clusters and DotPlot plots of Eph and ephrin expression showed only one neuronal cluster (N26), likely from outside of RMS with sex-based differences. Neuroblast and astrocyte clusters had no observable sex differences in Eph and ephrin expression (Supplementary Figures 2B–D).

### 3.4 Extensive signaling interactions link neuroblast and astrocyte subclusters

The complex heterogeneity of Eph/ephrin expression patterns within neuroblasts led us to investigate potential Eph/ephrin-mediated cell–cell communication involving both homotypic and heterotypic interactions between neuroblast and astrocyte populations. We used CellChat (Jin et al., 2021) to model signaling interactions across the ages (P6, P12, and P60) (Figure 2E). All possible Eph and ephrin interactions are summarized using aggregate circle plots to highlight the strength (line thickness) and direction (arrow color) of signaling between neuroblast and astrocyte clusters at each age. These analyses revealed various homotypic and heterotypic interactions between all clusters. While at all ages there was signaling involving most clusters, by P60 signaling involving neuroblast clusters NB4 and NB9 and maturing neuroblast cluster MNB21 dominated. This suggests that overall inter-cluster Eph and ephrin communication changes over development.

### 3.5 Eph and ephrin proteins are expressed by RMS neuroblasts and astrocytes throughout development

**Figure 3:**
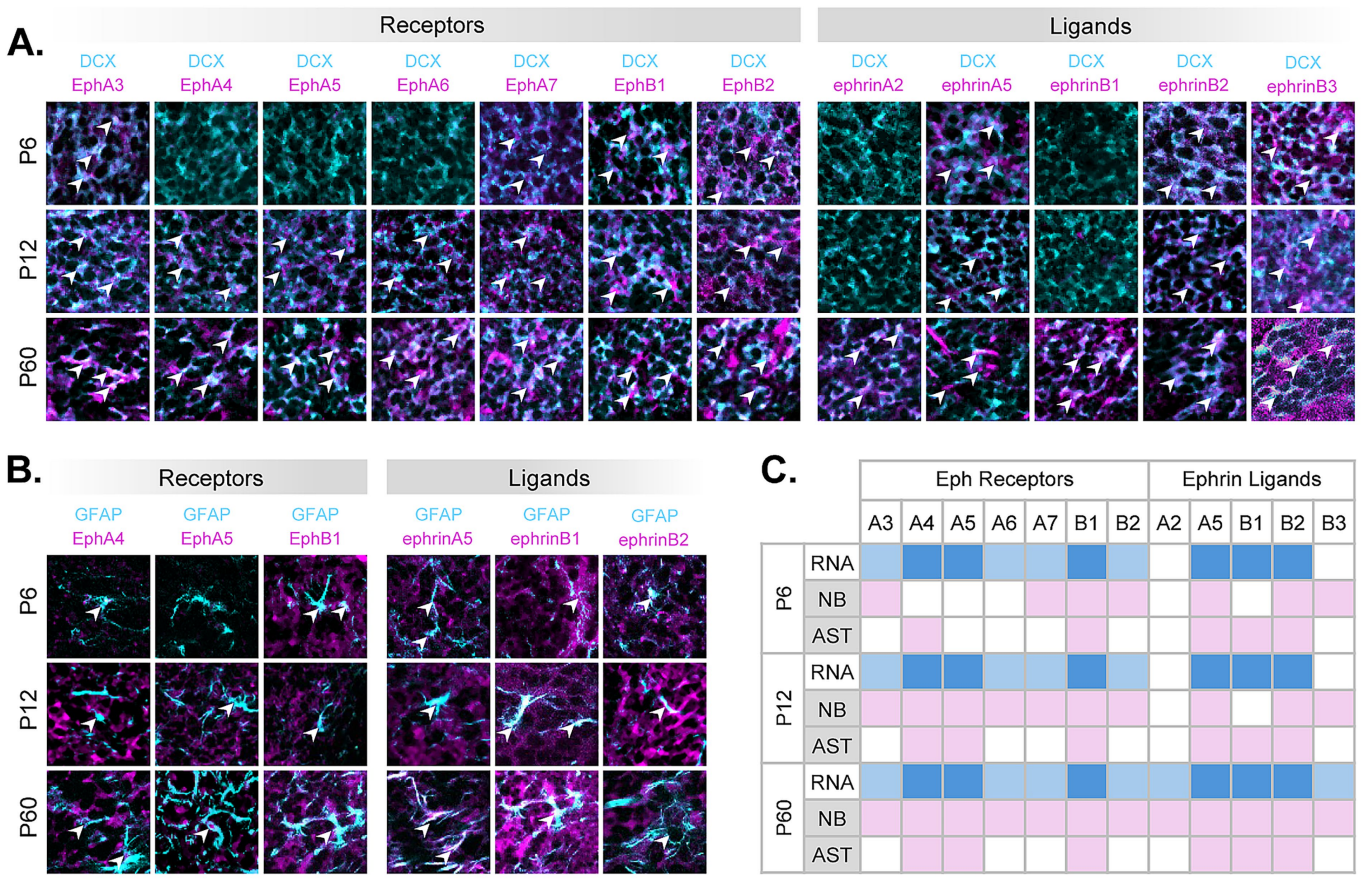
Confirmation of Eph/ephrin protein expression in RMS neuroblasts and astrocytes. Coronal sections of the RMS of P6, P12, and P60 mice show presence of select Eph receptors and ephrin ligands (magenta) on neuroblasts **(A)** and astrocytes **(B)** (DCX or GFAP, cyan). Arrowheads highlight representative co-expression within cell type. Scale = 50×50 μm. **(C)** RNA and protein expression summary table across ages. NB (neuroblasts), AST (astrocytes). Colors: light blue (RNA presence in neuroblasts), blue (RNA presence in both neuroblasts and astrocytes), and pink (protein presence).

Based on significant *Eph* receptor and *Efn* ligand RNA expression (Figure 2), we next wanted to validate which Eph and ephrins were present at the protein level in RMS neuroblasts and astrocytes. Since available Eph and ephrin antibodies (Supplementary Table 1) generally share a host species (rabbit), staining procedures were limited and did not allow for Eph/ephrin co-expression observations. We performed immunohistochemistry (IHC) on coronal slices of the anterior forebrain that contained the RMS at the three timepoints, P6, P12, and P60 (Figures 3A,B). Co-expression of DCX^+^ (neuroblasts) or GFAP^+^ (astrocytes) together with each of the significant *Eph* receptors or *Efn* ligands, based on scRNA-seq, was analyzed. Representative examples of co-expression of neuroblasts (cyan) or astrocytes (cyan) expressing Eph/ephrin antibodies (magenta) were labeled with white arrowheads (not all positive co-expressing cells are indicated). For P6 neuroblasts, our data confirmed the presence of EphA3, EphA7, EphB1, EphB2 receptors and ephrinA5, ephrinB2 ligands. In contrast to our scRNA-seq data, we saw additional expression of ephrinB3 (which showed low level expression in MNB13 and 21) but did not observe EphA4, EphA5, EphA6, or ephrinB1 in P6 neuroblasts. At P6, astrocytes were found around the periphery of the RMS; few were found within the RMS core. We captured these peripheral astrocytes and found that they expressed EphA4, EphB1 receptors and ephrinA5, ephrinB1, ephrinB2 ligands. Which was consistent with RNA data, except EphA5 expression was not detected (see Figure 3C, P6).

At P12, neuroblasts expressed an increased number of receptors (EphA3, EphA4, EphA5, EphA6, EphA7, EphB1, and EphB2) and ligands ephrinA5 as well as ephrinB2 and ephrinB3. At the P12 timepoint, protein expression was similar to RNA data, with the addition of ephrinB3 ligand expressed in neuroblasts. P12 astrocytes, which were now present within the RMS core, expressed EphA4, EphA5, EphB1 receptors and ephrinA5, ephrinB1, ephrinB2 ligands, which mimicked RNA expression profiles (see Figure 3C, P12).

At P60, we found a further increase in protein expression, with EphA3, EphA4, EphA5, EphA6, EphA7, EphB1, EphB2 receptors and ephrinA2, ephrinA5, ephrinB1, ephrinB2, ephrinB3 ligands expressed in neuroblasts. While astrocytes retained the same expression profiles as observed at P12. At P60, both neuroblast and astrocyte Eph/ephrin protein expression was consistent with RNA gene expression (see Figure 3C, P60).

These data indicated that while Eph and ephrin gene expression in astrocytes and neuroblasts observed by scRNA-seq stayed mostly consistent within subgroups and across ages (see Figure 1D), protein expression varied throughout development (Figure 3C), potentially indicating post-transcriptional regulation based on temporal environmental needs. These immunohistochemical studies helped us to narrow down Eph-ephrin signaling possibilities present in specific cell types (neuroblasts or astrocytes) and at each age group; however, which proteins were actively engaged in signaling were not captured.

### 3.6 Phosphorylation patterns indicate active Eph/ephrin signaling within the RMS

**Figure 4:**
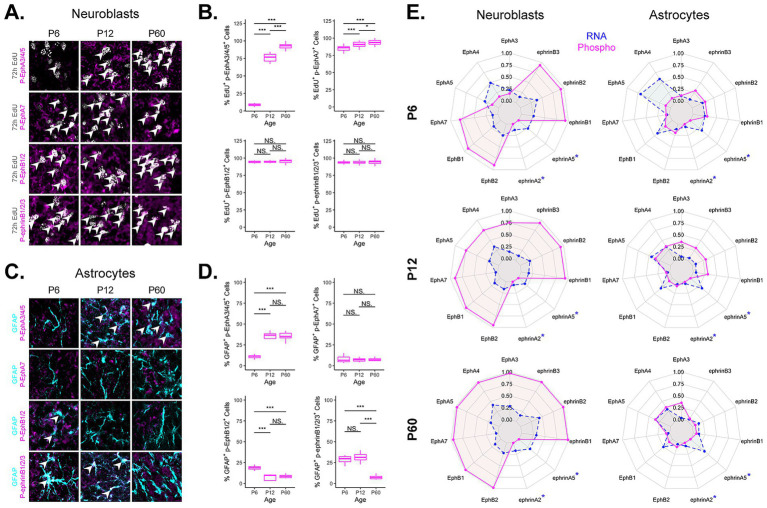
Quantification of Eph and ephrin phosphorylation across ages. **(A)** Representative images of RMS EdU^+^ neuroblast co-expression with phosphorylated Eph/ephrin antibodies at different ages. **(B)** Boxplots show percentage of EdU^+^ neuroblasts with phosphorylated Eph/ephrin co-expression at different ages. **(C)** Representative images of RMS GFAP^+^ astrocyte co-expression with phosphorylated Eph/ephrin antibodies at different ages. At P6 astrocytes are mainly found at the periphery of RMS. **(D)** Boxplots show percentage of EdU^+^ neuroblasts with phosphorylated Eph/ephrin co-expression at different ages. Statistical comparisons were performed using Wilcoxon rank-sum tests. Significance is indicated as follows: *p* ≤ 0.05 (*), *p* ≤ 0.01 (**), *p* ≤ 0.001 (***), *p* ≤ 0.0001 (****), ns (not significant). **(E)** Radar graphs showing differentially expressed Eph and Efn genes (blue) and phosphorylated proteins (magenta) in neuroblasts and astrocytes. Blue asterisk represents ligands incapable of phosphorylation. Each expanded concentric ring represents a 25% increase, with a maximum of 100% (outer ring) and a minimum of 0% (central point). *EphA6 is not included in this analysis due to the absence of antibodies to phosphorylated EphA6.

**Figure 5:**
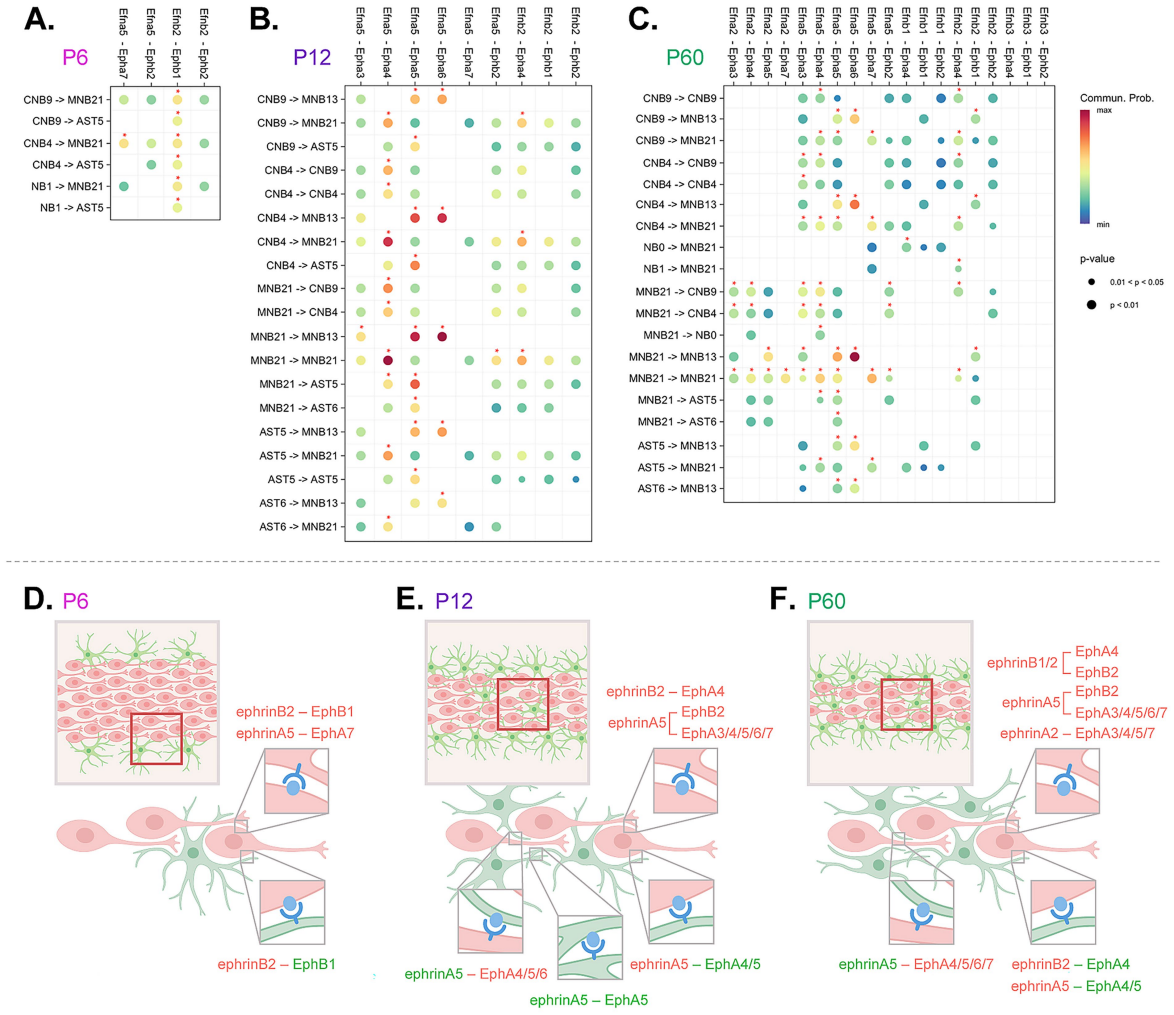
Integrated cell-to-cell Eph and ephrin communication summary across development. **(A–C)** Abridged CellChat DotPlot of Eph/ephrin ligand-receptor communication network between astrocyte (AST5, AST6) and neuroblast (cycling neuroblasts, CNB9, 4; neuroblasts, NB0, 1; maturing/mature neuroblasts, MNB13, 21) combinations. The dot color and size represent the calculated communication probability and *p*-values, respectively (*p*-values are computed from a one-sided permutation test). Communication probability above 0.01 threshold is denoted with a red asterisk. The *p*-value and communication probability legend applies to all CellChat DotPlot panels. **(D–F)** Schematic summary of signaling pairs including homotypic and heterotypic interactions between neuroblasts (red) and astrocytes (green). Red boxes outline depicted region within the RMS, where predicted interactions take place. Eph receptors are shown in blue and ephrin ligands are shown in light blue.

As the RMS undergoes a temporal reduction in cross-sectional area over time and the astrocytic meshwork within the RMS is increased, we projected that phosphorylation patterns for Ephs/ephrins would reflect these structural changes (Figure 4). To investigate Eph/ephrin activation status within the RMS across developmental stages, we examined expression of phosphorylated Ephs/ephrins using phospho-antibodies on forebrain sections containing the RMS. Since IHC experiments in Figure 3 informed us as to which Eph or ephrin proteins were present in neuroblasts or astrocytes, we could analyze Eph/ephrin signaling based on specific subgroups (i.e., EphA3/4/5/7, EphB1/2, EphrinB1/2/3). This was necessary since individual phosphorylated Eph/ephrin antibodies that are suitable for IHC are limited. Therefore, we used a combination of individual and compound antibodies. Additionally, to label newly generated neuroblasts, we used a 72 h EdU labeling scheme together with DCX co-labeling to identify migratory neuroblasts. Antibodies to phosphorylated receptors EphA3/4/5 (Tyr779, Tyr833); EphA7 (Tyr614); EphB1/2 (Tyr594, Tyr604) were used (no phospho-antibodies were available for EphA6). We used phosphorylated ephrinB antibody (ephrinB1/2/3(Tyr324)). Only the ephrinB group of ligands is capable of phosphorylation (Gerstmann and Zimmer, 2018), therefore, EphrinA ligands were not included in these calculations since they lack an intracellular domain and are not phosphorylated (Gerstmann and Zimmer, 2018). EphrinA interactions will be detailed in the following sections based on the presence of RNA, protein, and high communication probability (see Figure 5).

At P6, we found very low levels of phospho-EphA3/4/5 receptors in EdU^+^ neuroblasts (≈9%), but that changed by P12 with a dramatic increase (≈78%) and a further increase by P60 (≈91%) (Figures 4A,B). Whereas phospho-EphA7 in neuroblasts remained widely expressed at or above 86% across all ages but had a significant increase from P6 to P12 and then a smaller increase at P60 to ≈94% (Figures 4A,B). While phospho-EphB1/2 and its potential ligands, phospho-ephrinB1/2/3 remained consistently high at or above ≈94% in all neuroblasts across all timepoints (Figures 4A,B). This suggests that active signaling via neuroblast-neuroblast interactions involving ephrinB1/2/3-EphB1/2 and ephrinA-EphA7 pairs were maintained across all developmental timepoints.

For GFAP^+^ astrocytes (Figures 4C,D), we found that at P6 phospho-EphA3/4/5 co-expression was low at ≈11% but it increased significantly at P12 to ≈35% at which point it remained constant to P60. Phospho-EphB1/2 expression started off at ≈19% and then dropped to under 10% at both the P12 and P60 timepoints. Phospho-EphA7 remained consistently low at all ages (under 8%). Phospho-ephrinB1/2/3 at P6 showed co-expression with ≈30% of astrocytes and that increased slightly to ≈31% at P12 but then dropped to ≈7% at P60.

It is important to note that neuroblast-neuroblast interactions predominated at P6 and persisted through P12 and P60 even as astrocytes infiltrate the RMS. In contrast, EphA3/4/5 signaling increased in both neuroblasts and astrocytes as the RMS matured, correlating with increased astrocyte presence in the RMS core. Higher EphB1/2 and ephrinB1/2/3 signaling in astrocytes at P6 may be related to active astrocyte rearrangement and integration within the RMS core, whereas low signaling at P60 could reflect a stabilized astrocyte architecture not present earlier in development.

### 3.7 Eph/ephrin RNA expression correlates with activated Eph/ephrin signaling in neuroblasts

To compare ligand and receptor presence at both the transcriptional and post-translational, activated levels, we generated radar charts displaying scaled expression values of Eph receptors and ephrin ligands measured by either scRNA-seq or phosphorylation IHC (Figure 4E), where expression was scaled from 0 to 1. Ephs and ephrins included on the plot were significantly present in at least one cell type (Supplementary Figure 4), confirmed by protein presence (Figure 3), and showed up in at least one cell–cell interaction (Supplementary Figure 5). Efna2 and Efna5 are incapable of phosphorylation and are denoted with a blue star, indicating phosphorylation data were not possible. RNA expression was indicated in blue and phosphorylation co-expression in either neuroblasts or astrocytes was indicated in magenta. These plots highlight that while RNA expression in neuroblasts and astrocytes was a relatively good predictor for Eph/ephrin activity, it does not always match phosphorylation patterns throughout development. Specifically, at P6 both neuroblasts and astrocytes show the greatest variation between RNA expression and protein phosphorylation. These studies demonstrate the importance of combining both RNA and protein data to interpret potential activation of signaling pathways.

### 3.8 Major Eph/ephrin signaling pairs in the RMS shift with age

In complex systems, such as Eph/ephrin signaling where multiple signaling possibilities are conceivable based on multiple signaling partners, there is a need for predictive modeling. We used an integrated approach to narrow down and identify significant Eph receptor-ephrin ligand interactions across development in the RMS. We used CellChat to identify relative contribution pairings between Eph receptors and ephrin ligands (Figure 5A–C). We applied a communication probability threshold of ≥ 0.01 together with permutation-based *p*-value filtering (*p* < 0.05) ensuring statistical robustness (Jin et al., 2021). Interactions above this threshold, considered high confidence, were visualized on a dot plot and highlighted with an asterisk. Then based on the above studies that identified presence of protein (Figure 3) and phosphorylation status (Figure 4), we narrowed down interactions that were most likely (Figures 5A–C). To summarize findings within each of the timepoints, we created a conceptual model (Figures 5D–F) highlighting the directionality of signaling between neuroblast-neuroblast, neuroblast-astrocyte and astrocyte-astrocyte, identifying key ligand and receptor expression networks across subpopulations based on our phosphorylation data.

At P6 (Figures 5A,D), the majority of predicted significant interactions were through ephrinB2-EphB1, and ephrinA5-EphA7. EphrinA5-EphA7 occurred in neuroblast-neuroblast interactions between mature cluster MNB21 and cycling cluster CNB4. EphrinB2-EphB1 communication was found between all types of neuroblast clusters (CNB4, CNB9, NB1, MNB13, MNB21), and was also prominent in astrocyte-neuroblast interactions between astrocyte cluster AST5 and cycling neuroblast clusters CNB4, CNB9, and NB1. This suggested extensive Eph-ephrin binding between neuroblasts, the major cell type occupying the RMS at P6, and interactions between neuroblasts and astrocytes that border the RMS.

At P12 (Figures 5B,E), in addition to the interactions occurring at P6, new communication pairings mediated through EphA5, EphA4, and EphA3 receptors were predicted. EphrinB2-EphA4, ephrinA5-EphA4, and ephrinA5-EphA5 interactions between cycling (CNB9, CNB4) and mature clusters (MNB13, MNB21) were predicted. MNB21 was also involved in unique intrinsic ephrinB2-EphA4 mediated interactions as well as ephrinA5-EphA3 interactions with MNB13. Astrocyte (AST5, AST6)–neuroblast (CNB9, CNB4, MNB13, MNB21) interactions were predicted through ephrinA5-EphA4 and ephrinA5-EphA5, while astrocyte-astrocyte interactions were only possible through ephrinA5-EphA5. Although at this age *Epha7* RNA was expressed at lower levels and the predicted interaction did not meet the significance threshold in our CellChat communication probability analysis, we observed clear evidence of protein expression and phosphorylation, underscoring the importance of validating signaling activity at the protein level. These signaling shifts suggest a developmental change in neuroblast and astrocyte signaling needs, likely due to RMS reorganization—the RMS area decreases and astrocytes now make up the astrocytic network that aids neuroblast migration (Figure 2). An increase in cell–cell contact would create more possibilities for Eph/ephrin interactions.

At P60 (Figures 5C,F), in addition to the above P12 pairings, ephrinB1 and ephrinA2 mediated interactions now possible. EphrinA2-EphA5 showed a unique signaling occurrence between a maturing cluster (MNB13) and mature cluster (MNB21), which was not found at earlier timepoints. EphrinA2-EphA7 interaction also uniquely occurred within mature cluster (MNB21), which also showed ephrinA2-mediated signaling through EphA3/4 on cycling neuroblasts (CNB4, CNB9). This could indicate emerging signaling distinctions for more mature neuroblasts at the older timepoint. There were no significant astrocyte-astrocyte interactions through Eph-ephrin signaling at this age, suggesting that once the cytoarchitecture is set at P12, the need for homotypic astrocyte-astrocyte interactions through Ephs and ephrins may be eliminated. The overall addition of predicted signaling pairs based on increasing age suggests that as the pathway develops and is further refined there was an increase in signaling pairs to reinforce the RMS. These analyses indicated that Eph-ephrin signaling between astrocytes and neuroblasts is dynamic and cluster-specific from P6 to P60. These data also supported previous findings on the importance of the EphA4 receptor, as we see that EphA4-mediated signaling emerges by P12 and is predicted for neuroblast-neuroblast and neuroblast-astrocyte interactions through P60.

## 4 Discussion

To investigate regulatory control of the temporally dynamic, robust forebrain neuroblast migration pathway, the RMS, we focused on the Eph-ephrin signaling system. Although individual Eph-ephrin pathways have been linked to neuroblast migration through the anterior forebrain, the temporal diversity and full repertoire of possible interactions have not been systematically investigated. We first focused on cell-specific RNA transcript expression patterns and then validated these findings at the protein and phosphorylated (activated) protein level. Through this approach, we identified core signaling networks that defined temporal and cell–cell interactions. Specifically, our studies indicated that active Eph-ephrin signaling is present in RMS neuroblasts and astrocytes across all examined ages; however, signaling pairs are fluid and dependent on changing neuroblast-neuroblast and neuroblast-astrocyte organization within the temporally distinct RMS. By combining observations from the developing cytoarchitecture, single cell transcriptomics, Eph/ephrin activation, and ligand-receptor interactive modeling, we were able to streamline a complex signaling system and distinguish principal ligand-receptor interactions in neuroblasts and astrocytes of the RMS across development.

### 4.1 Cytoarchitectural changes

Postnatal neurogenesis in the V-SVZ with subsequent transportation of large quantities of neuroblasts through the anterior forebrain to the OB is a crucial developmental process that is maintained even as the surrounding brain regions mature. Strict regulatory control is required to ensure consistent delivery of new neurons to the OB to support functions such as circuit refinement and sensory processing involved with the sense of smell (Lledo et al., 2006; Ming and Song, 2011), even as the mouse ages and neurogenesis and neuroblast migration are scaled down (Obernier and Alvarez-Buylla, 2019; Shook et al., 2012). We showed at P6 that the RMS cytoarchitecture progressed from a loose aggregate of migrating neuroblasts with scattered astrocytes found at the RMS periphery, to a composite formation of neuroblast chains surrounded by astrocytes at P12, and then ultimately to a restricted, cell-dense array of migratory neuroblasts surrounded by a meshwork of astrocytes at P60. As the RMS pathway narrows from P6 to P60, a tightly regulated balance of neurogenesis and migration ensures continuous efficient delivery of new neurons into the olfactory bulb throughout the life of mouse.

### 4.2 RMS transcriptional changes

Neural stem cells in the V-SVZ consist of heterogeneous subpopulations based on their location of origin and are thought to be predetermined in their eventual interneuron specification (Cebrian-Silla et al., 2021; Fiorelli et al., 2015). As their progeny, neuroblasts, start their long-range migration toward the OB, they intermix and migrate uniformly in the RMS until they segregate to their final interneuron-specific destination within either the granular cell layer or periglomerular layer of the OB (Chaker et al., 2016; Fiorelli et al., 2015). Within the RMS, we found dividing (cycling), intermediate, and maturing neuroblast subpopulations at all three timepoints (P6, P12, and P60); however, the maturing populations’ proportions decreased with age. This age-related decline in the more mature neuroblast populations suggests a delay in differentiation within the RMS. Using the expression of genes associated with neural stem cell V-SVZ spatial origin (Cebrian-Silla et al., 2021; Fiorelli et al., 2015), we found similar profiles within the maturing neuroblast populations. Dividing them into two subtypes, one exhibiting lateral V-SVZ markers with a likely fate commitment to GABAergic interneurons (Calbindin^+^ and TH^+^), and the other exhibiting dorsal V-SVZ markers with a likely fate commitment to glutamatergic interneurons (Calretinin^+^). This would support lineage-directed predetermination in migratory neuroblasts; however, in intermediate neuroblast populations, in addition to defined populations expressing either lateral or dorsal V-SVZ markers, some intermediate subpopulations presented a mixture of these lineage markers, suggesting that a further delineation and understanding of fate specification at different neuroblast stages is needed.

### 4.3 *Eph*/*Efn* RNA signatures

Our group had previously shown the heterogeneity of RMS neuroblasts based on gene expression of *EphA4* receptor and all *Efns* (Todd et al., 2017). These initial studies focused on the role of EphA4 in the RMS and concluded that EphA4 likely works in concordance with other Eph receptors as not all neuroblasts were affected in EphA4^−/−^ mice (Todd et al., 2017). Here, we extended these original studies to include all Eph, as well as ephrin, subclasses and confirmed that *Eph*/*Efn* heterogeneity identifies specific neuroblast subpopulations. Our current results demonstrate that in addition to *EphA4*, expression of *EphA3*, *EphA5*, *EphA6*, *EphA7*, *EphB1*, *EphB2* receptors was found in neuroblast subpopulations, as well as *EphA3*, *EphB1* receptors in all astrocyte subpopulations. Specifically, we highlighted *Eph*/*Efn* expression differences between cycling, intermediate, and maturing/mature neuroblast subpopulations which may relate to discrete or redundant functional roles across development. To identify potential neuroblast-neuroblast *Eph*/*Efn* interactions, we calculated overall communication probabilities across ages and found extensive homotypic and heterotypic interactions at each timepoint (see Figure 1E). While this analysis provided valuable insight, scRNA-seq lacked protein-level resolution which we then addressed through complementary protein expression analysis.

### 4.4 Eph/ephrin protein expression and phosphorylation

We initially identified the presence of Eph/ephrin protein on RMS neuroblasts and astrocytes; however, to focus on potential signaling interactions we identified phosphorylated, activated forms of Ephs/ephrins in neuroblasts and astrocytes across developmental timepoints. This allowed us to refine and clarify cell-specific signaling networks. We found that active (phosphorylated) EphA3/4/5 signaling increased in both neuroblasts and astrocytes as the RMS matured. Now, in addition to EphA4’s known role in RMS regulation, additional active signaling pathways revealed that EphA3 and EphA5 were also involved. Interestingly, RMS disorganization due to absence of EphA4 did not occur until around P12 (Todd et al., 2017), a time when astrocytes become integrated within the RMS core. Indeed, we found low, to absent, phosphorylation levels of EphA3/4/5 signaling in neuroblasts at P6, indicating that a requirement for EphA3/4/5 occurs after P6, corresponding to establishment of astrocytic networks.

In contrast, we found phosphorylation of EphB1/2, ephrinB1/2/3, and EphA7 in neuroblasts occurred broadly across all ages, suggesting a continued requirement during RMS development (P6–P60). In astrocytes, EphB1/2 and ephrinB1/2/3 phosphorylation status was highest early in the development (P6) and then decreased greatly by P60, suggesting early roles in astrocyte organization before or during the time astrocytes initially become integrated within the RMS core. Low signaling levels at P60 could reflect a reduced need, due to a stabilized astrocytic network. By comparing RNA transcriptomics with active Eph/ephrin signaling across development, we demonstrated that while RNA is a predictor for potential signaling interactions, the presence of proteins and ultimately phosphorylated proteins validates which ligands and receptors actively participate in RMS regulatory control (see Figure 4E).

### 4.5 Integrated interactive modeling

Eph receptor-ephrin ligand communication network analyses (CellChat) allowed us to infer cluster specificity and directionality of signaling between RMS astrocytes and neuroblasts. Protein and specifically phosphorylation status helped us narrow down signaling pairs across P6, P12, and P60 timepoints. Although Ephs and ephrins can trigger diverse cell responses dependent on context (i.e., cell–cell specificity and microenvironment), previously characterized roles of specific ligand-receptor pairs provide a foundation to infer potential roles in our system.

At P6, we found only two major predicted signaling pairs: ephrinB2–EphB1 and ephrinA5–EphA7 (see Figure 5D). EphrinB2–EphB1 interactions are known to be involved in repulsion at cell boundaries and cell guidance (Smith et al., 1997; Teng et al., 2013). Based on our studies, peripheral astrocytes expressing EphB1 receptor may repulse migrating neuroblasts expressing ephrinB2, and since neuroblasts express both EphB1 receptor and ephrinB2 ligands contact-mediated repulsion may occur between neuroblast subtypes. Additionally, ephrinA5–EphA7 interactions are known to promote migration and adhesion (Nguyen et al., 2017), which would facilitate migration between EphA7-expressing maturing neuroblasts with ephrinA5-expressing cycling neuroblasts. At P6, neuroblasts were found within the RMS with astrocytes found mainly at the periphery, therefore Eph-ephrin signaling appears to be important for neuroblast-neuroblast facilitated migration and allows astrocyte navigation into the RMS core.

At P12, we observed significant additions of EphA6, EphA5, EphA4, EphA3, and EphB2 mediated interactions between neuroblasts and astrocytes (see Figure 5E). EphrinA5–EphA3 and ephrinA5–EphB2 have been previously linked with cell repulsive migration (Himanen et al., 2004; Shi et al., 2010) and were predicted between mature neuroblast clusters. Other predicted interactions involving ephrinA5–EphA6, ephrinA5–EphA5 and ephrinB2–EphA4 are known to promote cell adhesion (Deschamps et al., 2010; Nguyen et al., 2017; Poitz et al., 2015). EphrinA5–EphA5 interactions included both neuroblasts and astrocytes expressing both the receptor and ligand which resulted in neuroblast-neuroblast, neuroblast-astrocyte, and astrocyte-astrocyte signaling combinations that likely use adhesion to aid in chain migration, stabilization of neuroblast chains in astrocytic networks, and maintenance of network integrity. EphrinA5–EphA6 and ephrinB2–EphA4 may also aid chain formation between neuroblasts with ephrinA5–EphA6 also potentially facilitating neuroblast-astrocyte adhesion. Other neuroblast-neuroblast and neuroblast-astrocyte interactions were also predicted to occur through ephrinA5–EphA4 that could support astrocyte-neuroblast contact-dependent positional remodeling (Eberhart et al., 2004; Shu et al., 2016) a means to optimize migration.

At P60 the few additions of new signaling pairs included those mediated through ligands ephrinA2 and ephrinB1. EphrinB1–EphA4 interactions are known to promote cell adhesion (Murcia-Belmonte et al., 2025), and forward signaling through EphA4 receptors on mature neuroblasts may facilitate adhesion enabling dynamic remodeling of migrating neuroblast chains. EphrinA2 has been shown to play a role in cell differentiation (Homman-Ludiye et al., 2017) and in the RMS it was expressed in mature neuroblasts. EphrinA2–EphA7 has been implicated in negative regulation of proliferation (Jiao et al., 2008) and as this interaction is predicted to take place between mature neuroblast subpopulations it may reinforce and promote maturation. At P60, we do not see significant Eph-ephrin mediated interactions between astrocytes; however, neuroblast-astrocyte interactions persisted, suggesting their requirement in maintaining RMS integrity and migratory control.

Additionally, we found a neuron cluster (OBN18) with unique *Scgn* expression that has been implicated in RMS neuroblast migration (Hanics et al., 2017). After analyzing predicted Eph/ephrin CellChat interactions, we found significant communication between OBN18 neurons and RMS neuroblasts and astrocytes. Indicating that other cell types aid in regulatory control of the RMS.

While our studies focused mainly on canonical Eph/ephrin interactions, non-canonical interactions also exist. Ephs/ephrins have been shown to interact with integrins, glycoproteins (e.g., Reelin), and other receptor tyrosine kinases (e.g., FGFR1) (Bouché et al., 2013; Prydz et al., 2025; Sentürk et al., 2011). These factors all have known roles in migration. Integrins mediate extracellular matrix adhesion and integrin-Eph signaling may promote or inhibit detachment and influence directional movement (Arvanitis and Davy, 2008). Activation of FGFR1 in cells expressing EphB2 has been shown to inhibit cellular segregation and migration (Prydz et al., 2025) and we found *Fgfr1* expression in mature neuroblasts (MNB21) and astrocytes. Reelin can independently activate EphB receptors resulting in cytoskeletal changes (Bouché et al., 2013). Reelin can also bind to ephrinB ligands, which then associate at the membrane with Reelin receptors, leading to phosphorylation of adaptor protein Dab1 and accompanying cytoskeletal changes. Reelin has been described as promoting the switch from tangential to radial migration in the distalmost end of the RMS within the OB (Hellwig et al., 2012). However, we did not observe *Reln* expression in neuroblasts or astrocytes of the RMS, suggesting that Reelin’s function may be limited to within the OB but highlighting an area for future research.

## 5 Conclusion

We present studies that show Eph/ephrin signaling is dynamically regulated at the post-transcriptional level during RMS maturation, potentially orchestrating the timing of astrocyte structural transitions in response to developmental cues and the establishment of a mature RMS migratory pathway. Combining RNA-based datasets with assessment of phosphorylated (activated) Eph and ephrin signaling pathways allowed us to generate a refined model of this complex signaling system based on identifying key molecular players within the broader interactome (see Figures 5D–F). This integrative approach revealed functional relationships that may be overlooked in single-gene (or even combinatorial) knockout models, which often capture only partial aspects of signaling pathways. Together, these data provide a foundation for future investigations into the dynamics of Eph-ephrin signaling or other complex signaling systems. Understanding the regulation of migration control can provide insight into human congenital conditions, such as cortical malformations and neurodevelopmental disorders, and help identify strategies to restore or redirect migration in pathological contexts (Guerrini and Parrini, 2010).

## Data Availability

The scRNA-seq data generated for this study is deposited in the Gene Expression Omnibus (GEO) under accession number GSE306762.
