# Supplementary material for: Developmental regulation of long-range neuroblast migration by Eph/ephrin signaling
Source: Front Neurosci. 2025 Oct 8;19:1670635. doi: 10.3389/fnins.2025.1670635 (PMC12540379; doi:10.3389/fnins.2025.1670635)
Supplement: Supplementary file 1 [file Table_1.DOCX]

**Methods Supplement Table 1.** Primary antibodies information.

| Antibody | Host Species | Dilution | Supplier | RRID |
| --- | --- | --- | --- | --- |
| anti-GFAP | Goat | 1:500 | Thermo Fisher Scientific | AB_2942816 |
| anti-GFAP | Rat | 1:2000 | Thermo Fisher Scientific | AB_86543 |
| anti-DCX | Guinea pig | 1:1000 | Sigma-Aldrich | AB_2230227 |
| anti-DCX | Chicken | 1:1000 | Abcam | AB_2728759 |
| anti-PODXL | Mouse | 1:200 | Thermo Fisher Scientific | AB_2533411 |
| anti-pEphA3/4/5 | Rabbit | 1:200 | Thermo Fisher Scientific | AB_2554421 |
| anti-pEphB1/2 | Rabbit | 1:200 | Thermo Fisher Scientific | AB_2609042 |
| anti-pEphrinB1/2/3 | Rabbit | 1:200 | Thermo Fisher Scientific | AB_2815966 |
| anti-pEphA7 | Rabbit | 1:200 | Thermo Fisher Scientific | AB_2817082 |
| anti-EphA3 | Rabbit | 1:200 | Thermo Fisher Scientific | AB_2855098 |
| anti-EphA4 | Rabbit | 1:200 | Novus | AB_3244317 |
| anti-EphA5 | Rabbit | 1:200 | Proteintech | AB_2881289 |
| anti-EphA6 | Rabbit | 1:200 | Thermo Fisher Scientific | AB_2899823 |
| anti-EphA7 | Rabbit | 1:200 | Thermo Fisher Scientific | AB_2899824 |
| anti-EphB1 | Rabbit | 1:200 | Thermo Fisher Scientific | AB_2099828 |
| anti-EphB2 | Goat | 1:200 | Thermo Fisher Scientific | AB_2609043 |
| anti-EphrinA2 | Goat | 1:200 | Thermo Fisher Scientific | AB_2608569 |
| anti-EphrinA2 | Rabbit | 1:200 | Alomone Labs | AB_2756585 |
| anti-EphrinA5 | Rabbit | 1:200 | Alomone Labs | AB_2756586 |
| anti-EphrinB1 | Mouse | 1:200 | Thermo Fisher Scientific | AB_11219677 |
| anti-EphrinB2 | Rabbit | 1:200 | Thermo Fisher Scientific | AB_2854597 |
| anti-EphrinB3 | Rabbit | 1:200 | Thermo Fisher Scientific | AB_2533165 |

**Methods Supplement Table 1.** Secondary antibody information.

| Antibody | Host Species | Fluorophore | Dilution | Supplier | RRID |
| --- | --- | --- | --- | --- | --- |
| donkey anti-rat | Donkey | 405 | 1:500 | Thermo Fisher Scientific | AB_2890549 |
| donkey anti-goat | Donkey | 488 | 1:500 | Thermo Fisher Scientific | AB_2534102 |
| donkey anti-guinea pig | Donkey | 647 | 1:500 | Jackson ImmunoResearch Labs | AB_2340477 |
| donkey anti-mouse | Donkey | 546 | 1:500 | Thermo Fisher Scientific | AB_11180613 |
| donkey anti-rabbit | Donkey | 546 | 1:500 | Thermo Fisher Scientific | AB_2534016 |
| donkey anti-chicken | Donkey | 647 | 1:500 | Thermo Fisher Scientific | AB_2921074 |
