# Supplementary material for: Developmental regulation of long-range neuroblast migration by Eph/ephrin signaling
Source: Front Neurosci. 2025 Oct 8;19:1670635. doi: 10.3389/fnins.2025.1670635 (PMC12540379; doi:10.3389/fnins.2025.1670635)
Supplement: Supplementary file 2 [file Data_Sheet_1.PDF]

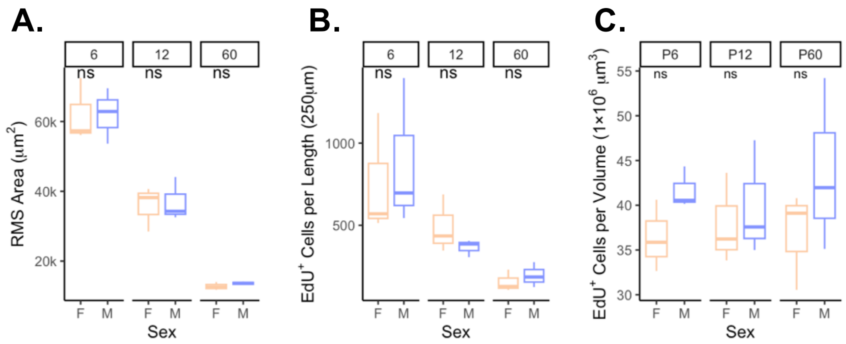

Supplementary Figure 1. Boxplots showing sex comparisons between female (peach), and male (blue) mice across P6, P12, P60 ages.

(A) Sex comparison for RMS cross-sectional area. (B) Sex comparison for total EdU^+ cells per 250μm length of the RMS. (C) Sex comparison for EdU^+ cell density per $1x10^6 μm^3$ RMS volume. Pairwise comparisons were performed using unpaired, two-sided Student's *t*-tests. Significance is indicated as follows: $p \leq 0.01$ (**), $p \leq 0.001$ (***), $p \leq 0.0001$ (****), ns (not significant).

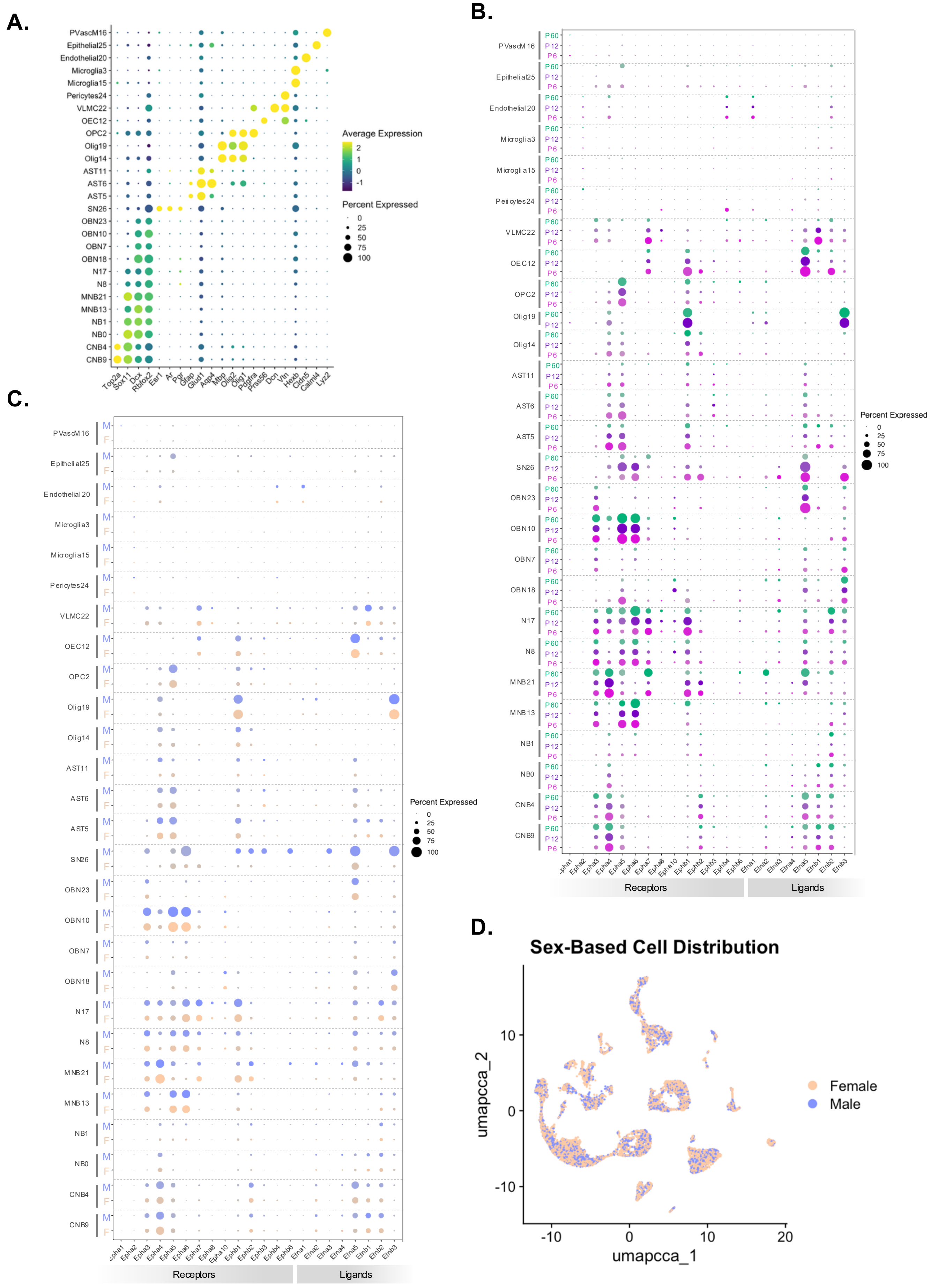

Supplementary Figure 2. Single-cell RNA-seq analysis of the micro-dissected RMS from P6, P12, and P60 mouse brains.

(A) DotPlot of cell-type specific marker expression. Abbreviations: cycling neuroblasts (CNB), neuroblasts (NB), maturing/mature neuroblasts (MNB), neurons (N), olfactory bulb neurons (OBN), hormonally affected neurons (SN), astrocytes (AST), oligodendrocytes (Olig), oligodendrocyte progenitor cells (OPC), olfactory ensheathing cells (OEC), vascular leptomeningeal cells (VLMC), perivascular macrophages (PVascM). (B) DotPlot of all clusters with Eph/ephrin expression by age: P6(pink), P12(purple), P60(green). (C) DotPlot of all clusters with Eph/ephrin expression by sex: male (blue), female (peach). (D) UMAP of sex-based cell distribution across clusters.

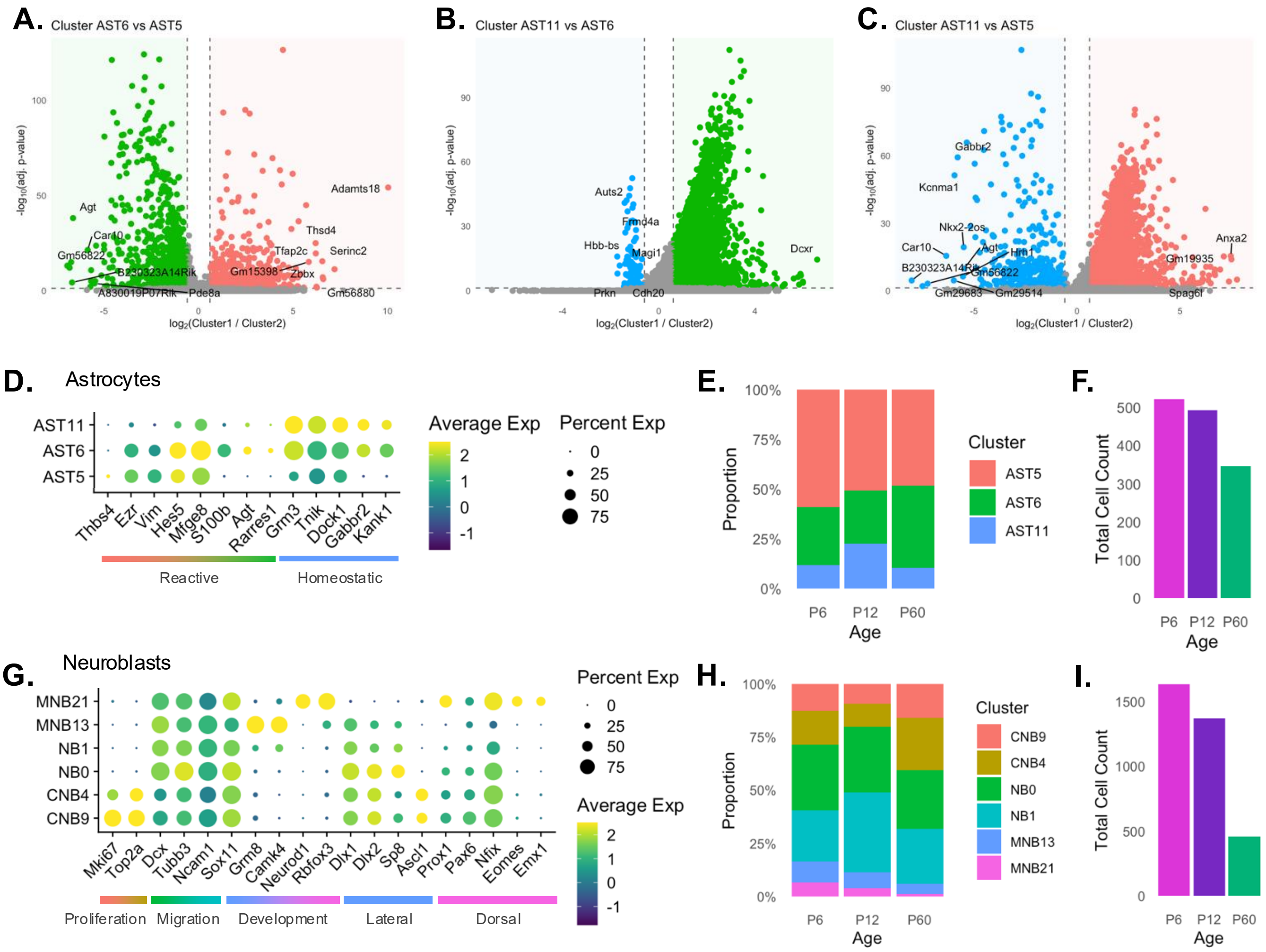

Supplementary Figure 3. scRNA-seq reveals astrocyte and neuroblast subpopulations.

(A-C) Volcano plot of significant differentially expressed genes in astrocyte clusters AST6 vs AST5, AST11 vs AST6 (B), AST11 vs AST5 (C). Colors: AST5 (red), AST6 (green), AST11 (blue). (D) DotPlot of astrocyte clusters with known reactive or homeostatic genes. (E) Quantitative representation of proportion of different astrocyte clusters at different ages. (F) Quantitative representation of overall cell numbers for all astrocyte clusters at different ages. (G) DotPlot of neuroblast clusters [cycling neuroblasts (CNB9,4), neuroblasts (NB0,1), maturing/mature neuroblasts (MNB13,21)] based on genes associated with specific cell processes and genes linked with V-SVZ location. (H) Quantitative representation of the proportion of different neuroblast clusters at different ages. (I) Quantitative representation of the overall cell numbers for all neuroblast clusters at different ages.

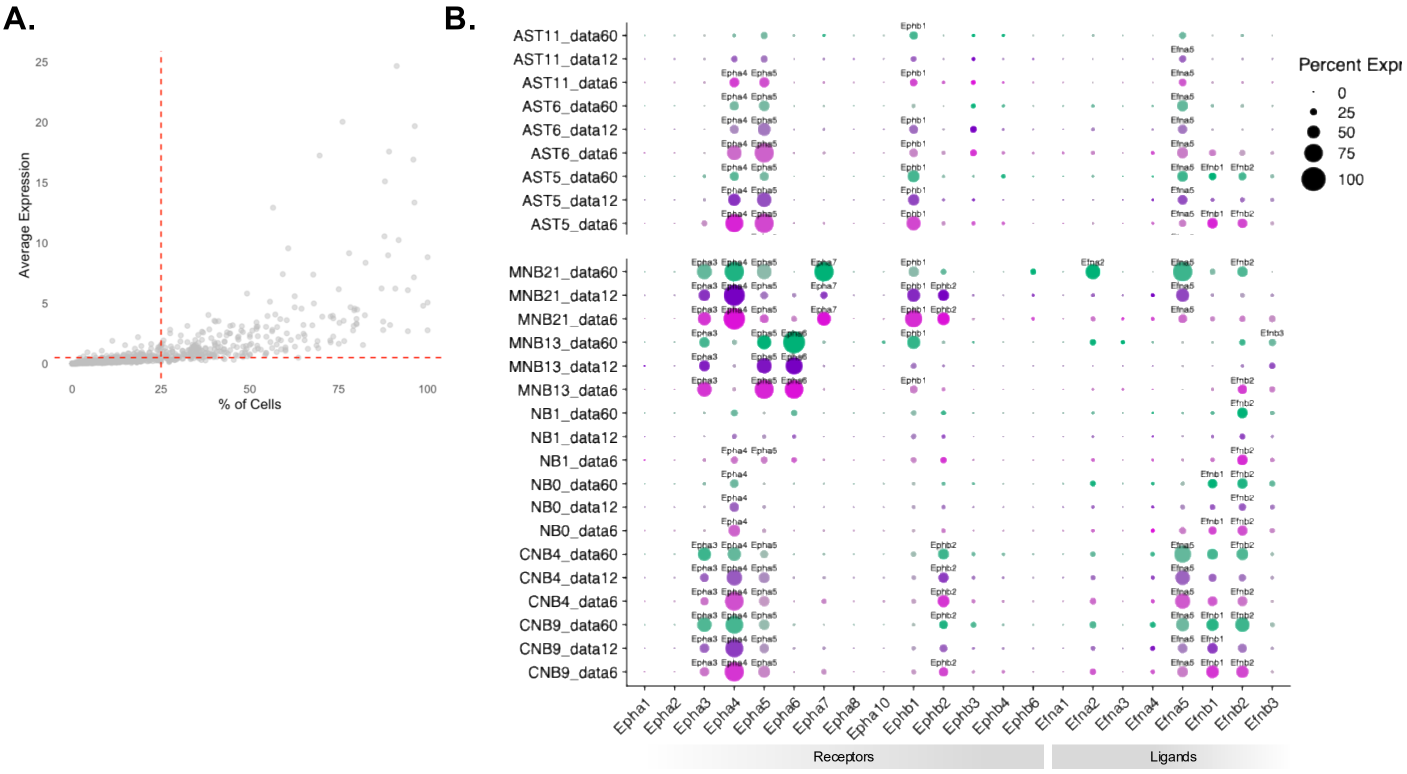

Supplementary Figure 4. Significance threshold identification.

(A) Scatterplot of average gene expression versus percent of cells expressing genes. Red dotted line represents thresholds of >25% cells expressing genes with an average expression >0.5. (B) DotPlot of Eph/ephrin expression in neuroblast and astrocyte clusters split by age P6(pink), P12(purple), P60(green). Significant expression is annotated with the gene name.

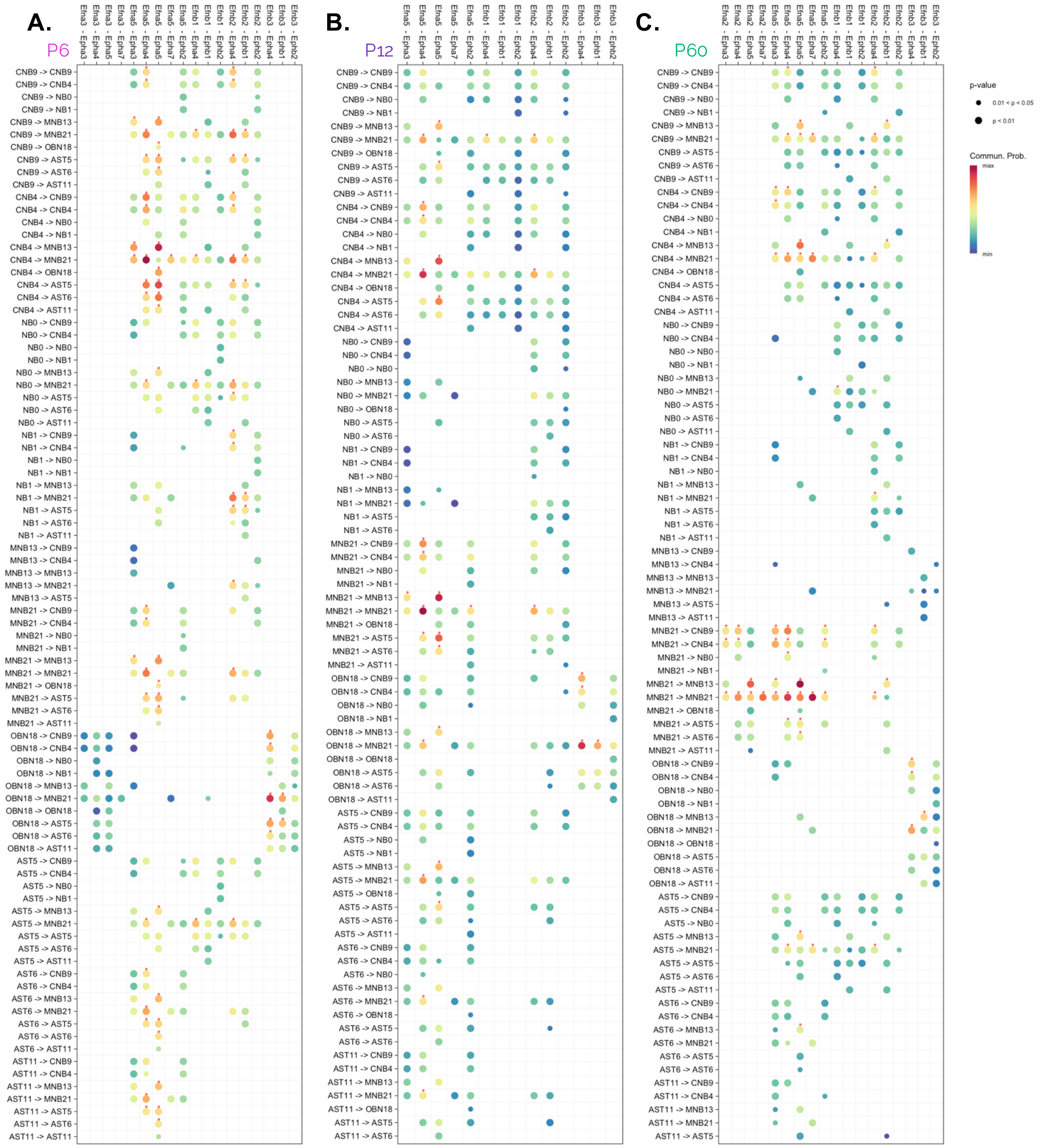

Supplementary Figure 5. Signaling summary of Ephs and ephrins at P6 (A), P12 (B), P60 (C).

(**A, B, C**) Full CellChat DotPlots of Eph/ephrin ligand-receptor communication network including homotypic and heterotypic interactions between astrocyte (AST5, AST6, AST11), neuroblast (cycling neuroblasts, CNB9,4; neuroblasts, NB0,1; maturing/mature neuroblasts, MNB13,21), and neuron (OBN18) clusters. The dot color and size represent the calculated communication probability and p-values respectively (p-values are computed from one-sided permutation test). The p-value and communication probability legend applies to all panels.
